# Maternal energy insufficiency affects testicular development of the offspring in a swine model

**DOI:** 10.1038/s41598-019-51041-y

**Published:** 2019-10-10

**Authors:** Yan Lin, Xue-Yu Xu, De Wu, Hao Lin, Zheng-Feng Fang, Bin Feng, Sheng-Yu Xu, Lian-Qiang Che, Jian Li, Yong Zhuo, Cai-Mei Wu, Jun-Jie Zhang, Hong-Jun Dong

**Affiliations:** 1 Key Laboratory for Animal Disease Resistance Nutrition of the Ministry of Education of China, Institute of Animal Nutrition, Sichuan Agricultural University, Chengdu, Sichuan 611130 China; 2 Key Laboratory for Neuro-Information of Ministry of Education, School of Life Science and Technology, Center for Informational Biology, University of Electronic Science and Technology of China, Chengdu, 610054 China; 3 School of Life Sciences, Sichuan Agricultural University, Ya’an, Sichuan 625014 China

**Keywords:** Genome-wide analysis of gene expression, Reproductive biology

## Abstract

We determined the effects of insufficient maternal energy on testicular development in offspring in a swine model. Thirty-six sows were divided into control (CON) and low-energy diet (LE) groups during gestation. We observed that the number of Sertoli, germ, and Leydig cells in the offspring of the CON group were significantly higher than those in the LE group at 28 and 120 d after birth. Furthermore, the percentage of apoptotic testis cells was significantly higher in the offspring of the LE group than in the CON group. Transcriptome analysis of differentially expressed mRNAs and long noncoding RNAs in offspring testes indicated that these RNAs were mainly involved in lipid metabolism, apoptosis, cell proliferation, and some pivotal regulatory pathways. Results revealed that AMPK–PI3K–mTOR, MAPK, and oxidative phosphorylation signaling pathways play an important role in mediating the programming effect of insufficient maternal energy on testicular development, and that this effect occurs mainly at an early stage in life. mRNA and protein expression analyses confirmed the importance of certain signaling pathways in the regulation of testicular development. This study provides insights into the influence and possible mechanism underlying the effect of inadequate maternal energy intake on testicular development in the offspring.

## Introduction

Some studies have shown that fetal organ development, growth, and metabolism, during gestation are critical^1,2^, and are significantly influenced by maternal nutrition during pregnancy^3–5^. Using a swine model, we have shown that the offspring of mothers fed a high-fat diet experience deterioration in ovarian health via the induction of oxidative stress and accelerated apoptosis^6^, and a high-fat, high-sucrose diet during pregnancy has been reported to put male offspring at a greater risk of infertility^7^. Meanwhile, changes in intrauterine nutrition have significant effects on the secretion of sex hormones, and on the development of sexual organs in female rats and hamsters, and in male as well as female lambs^8–10^. Importantly, the amount of research into the influence of maternal nutrition on metabolism or growth performance vastly outweighs that on reproduction, especially regarding maternal overnutrition or obesity. Hence, very little is known about the effects of maternal energy deficiency on male offspring reproduction and the possible associated mechanism.

Studies indicate that postnatal reproductive ability may be largely determined by the appropriate development of reproductive-function-related organs during fetal life. Vanbillemont *et al*.^11^ found that in humans, the birth weight positively correlates with plasma testosterone concentrations, and that reduced birth weight in males may be considered as an indicator of an increased risk of infertility^12^. Studies on male mammals suggest that a low maternal energy level can reduce the testicular weight and the number of Sertoli cells in Merino rams^13^. Rae found that a low maternal energy level has no effect on testicular weight, but the mRNA expression of *STAR* (steroidogenic acute regulatory protein) was significantly higher in sheep testes^14^. Research has shown that maternal selenium intake in goats^15^ and nutrition levels in cattle^16^ can affect testicular development and spermatogenesis in the male offspring by modulating testosterone synthesis and expression of relevant genes. Meanwhile, maternal undernutrition may be a significant factor in altering the reproductive potential of the progeny in sheep by reducing the pituitary activity^17^. Collectively, these studies suggest that exposure to an adverse maternal metabolic environment can impact male offspring, and that this phenomenon in turn can affect the metabolic and reproductive health.

Currently, very little is known about the mechanism through which the testicular development of male offspring is affected by inadequate maternal energy. To reveal the histological features and molecular mechanisms that underlie the effect of early-life energy on offspring testicular development and reproduction, we performed a histological comparison by performing hematoxylin-eosin (HE) staining of testicular paraffin sections and analyzed the number of germ cells and histological parameters. Furthermore, we conducted transcriptome analyses of the testes of offspring boars by high-throughput RNA sequencing (RNA-seq, which includes long noncoding RNA [lncRNA] sequencing), thus identifying differentially expressed genes (DEGs) and the affected signaling pathways. These results shed new light on the relationship between maternal energy and offspring testicular development, and provide guidance regarding animal feed formulas and human nutrition during pregnancy.

## Results

### Testicular development and morphological features of offspring boars

**Figure 1:**
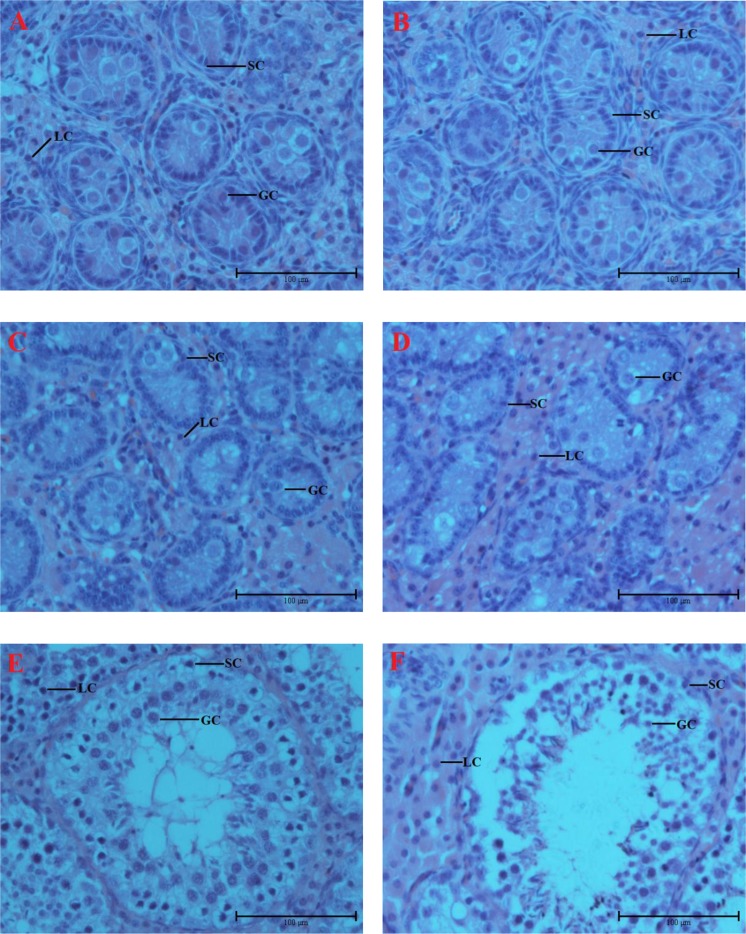
HE staining of testicular tissues in LE and CON groups on 0 d, 28 d, and 120 d. Testes of 0 d, 28 d, 120 d CON (**A**,**C**,**E**) and LE (**B**,**D**,**F**) group boars, respectively. Magnification: 400×. LC, Leydig cell; SC, Sertoli cell; GC, germ cell.

As shown in Table 1, lower maternal energy intake during pregnancy significantly influenced the offspring body weight at 0 and 28 d after birth and the testicular weight of the offspring boars at birth (0 d; P < 0.05), but had no effect at 120 d (P < 0.05). The structure of the testes is presented in Fig. 1. At 0 d, few germ and Sertoli cells were observed in the LE and CON groups, the structure of the spermatogenic cells was not different between the offspring of the CON and LE groups. Compared with the CON group at 28 d, more tubules were incomplete, the interstitial space between seminiferous tubules was larger, and fewer Leydig cells were apparent within the interstitial space in the LE group. However, compared with 0 d, the number of testicular cells slowly increased and no differentiated germ cells were observed. Besides, the number of testicular cells in the seminiferous tubules were not significantly different between the LE and CON groups at 28 d. At 120 d, the structure of the seminiferous tubules appeared more loosely in the LE group than in the CON group, and the interstitial space apparently increased. In addition, abundant vacuoles were observed in the seminiferous tubule cells in the LE group, which also had lower cell numbers, but compared with 28 d, larger testicular cells were observed in the seminiferous tubules, and differentiated germ cells were observed at 120 d. Thus, boars had already reached puberty at 120 d, and maternal malnutrition had a greater influence on the development of testicular tissue at 28 and 120 d.

**Table 1:** Effect of maternal energy restriction during gestation on testis weight and histology structure in offspring of boars (Mean ± SD).

Item	Time	LE	CON	P-value
Birth weight(kg)	0d	1.05 ± 0.35	1.44 ± 0.11	<0.01
Weaning weight(kg)	28d	4.88 ± 1.58	6.78 ± 1.49	0.016
Final weight(kg)	120d	50.19 ± 10.79	53.87 ± 7.95	0.40
Testicular weight(g)	0d	0.15 ± 0.02	0.24 ± 0.05	0.02
	28d	6.43 ± 1.54	6.67 ± 0.16	0.81
	120d	92.63 ± 35.78	92.03 ± 23.39	0.97
No. Sertoli cells per tubule	0d	21.22 ± 6.41	22.20 ± 3.38	0.06
	28d	23.97 ± 4.58	28.44 ± 5.63	<0.01
	120d	30.69 ± 9.73	37.04 ± 6.31	<0.01
No. Germ cells per tubule	0d	1.91 ± 0.65	2.01 ± 0.81	0.36
	28d	2.20 ± 0.59	2.43 ± 0.94	0.09
	120d	77.45 ± 20.81	101.88 ± 20.51	<0.01
No. Leydig cells per intertubule	0d	6.25 ± 1.41	9.86 ± 1.87	<0.01
	28d	12.48 ± 1.93	13.16 ± 1.82	0.11
	120d	37.71 ± 4.01	44.44 ± 2.88	<0.01
Diameter(µm)	0d	55.63 ± 5.21	54.58 ± 5.64	0.11
	28d	79.02 ± 8.86	76.95 ± 10.24	0.08
	120d	154.18 ± 15.57	167.87 ± 21.59	<0.01
No. Daily sperm production per tubule	120d	13.52 ± 5.07	17.19 ± 6.00	<0.01

No. Daily sperm production per tubule were predicted daily sperm production in seminiferous tubules at 120 d. n = 5 for each group.

We measured the diameter of seminiferous tubules in the LE and CON groups. At 0 and 28 d, no differences in the diameters of the seminiferous tubules were detected. At 120 d, the diameters of seminiferous tubules in the CON group were significantly greater than those in the LE group (Table 1, P < 0.01). Besides, the diameter of the seminiferous tubules in the two groups rapidly increased with body growth. The results revealed that these diameters in the CON and LE groups stably increased from birth to puberty, and that the development of the CON group was faster than that of the LE group.

At 0 d, few testicular cells were found in LE and CON boar testes, but the number of Leydig cells was significantly different between the two groups (Table 1, P < 0.01); At 28 d, only the number of Sertoli cells was significantly different (Table 1, P < 0.01). At 120 d, the numbers of Sertoli, germ, and Leydig cells in the CON group were significantly greater than those in the LE group (Table 1, P < 0.01), and the numbers of germ and Leydig cells increased rapidly from 28 d to 120 d. The sperm count in the seminiferous tubes at 120 d revealed that the estimated number of spermatozoa per day was higher in the CON group than in the LE group (P < 0.01).

### Testicular proliferation and apoptosis in offspring boars

**Figure 2:**
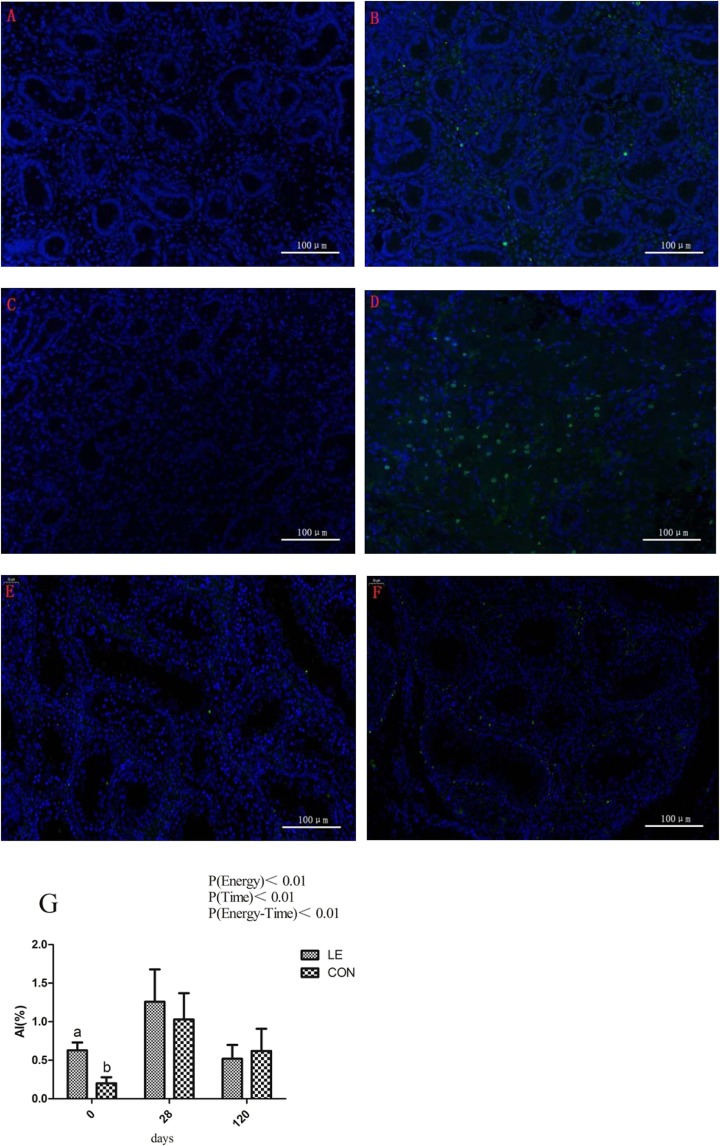
Effect of maternal energy restriction during gestation on apoptosis in testes in boar offspring. LC, Leydig cell; SC, Sertoli cell; GC, germ cell. (**A**,**C**,**E**) 0 d, 28 d, 120 d testes in the CON group boar, respectively; (**B**,**D**,**F**) 0 d, 28 d, 120 d testes in the LE group boar, respectively. Magnification: 200×. (**G**) The AI of LE and CON at different ages. AI = Apoptotic Index; a, b Means in rows with different superscript letters are significantly different (P < 0.05).

**Figure 3:**
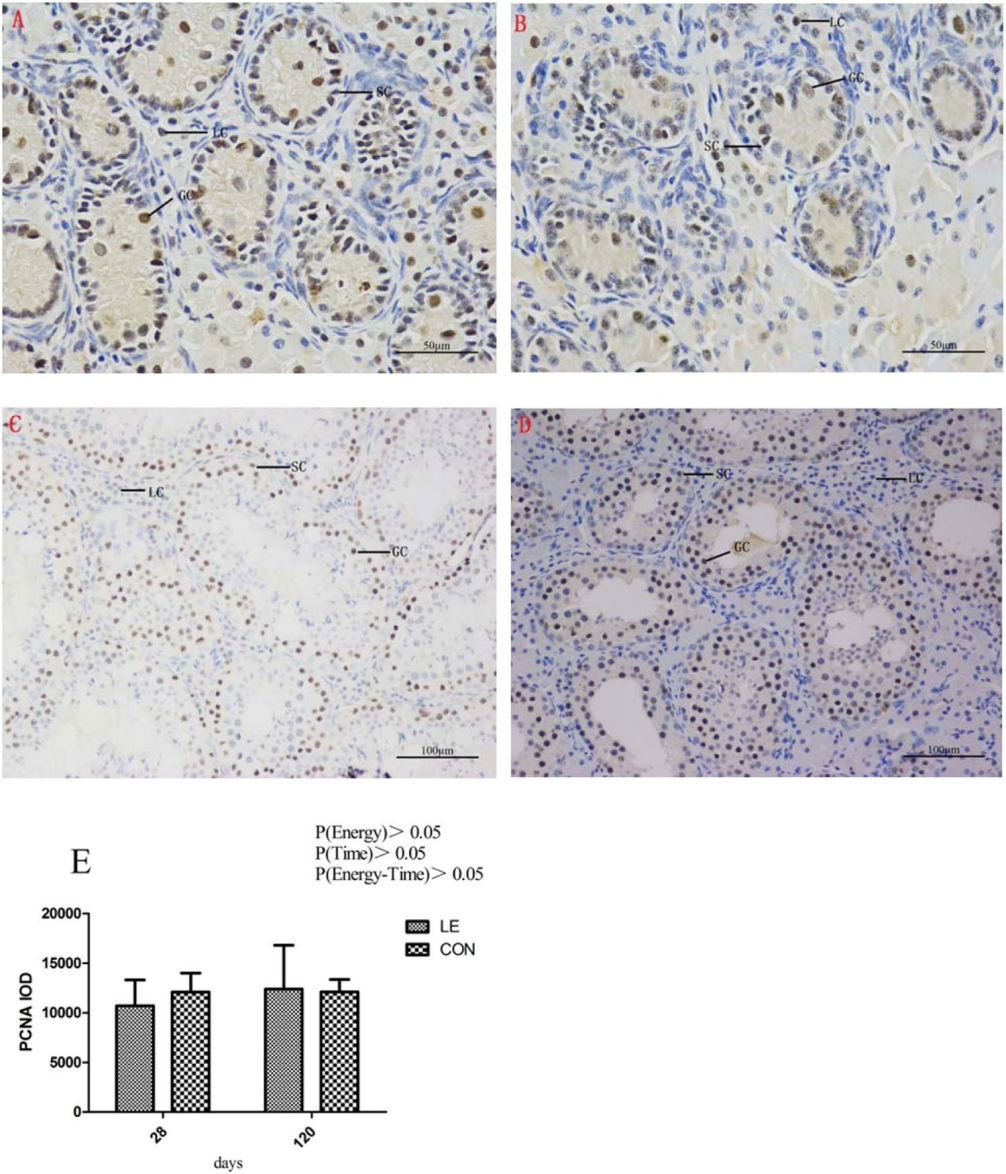
Effect of maternal energy restriction during gestation on testis cell proliferation in boar offspring. LC, Leydig cell; SC, Sertoli cell; GC, germ cell. (**A**,**C**) 28 d, 120d testes in the CON group boar; (**B**,**D**) 28 d, 120d testes in the LE group boar. Magnification: A and B, 400×; (**C**,**D**), 200×. (**E**) The PCNA IOD of LE and CON at different ages. PCNA = Proliferating Cell Nuclear Antigen, IOD = Integrated option density; a, b Means in rows with different superscript letters are significantly different (P < 0.05).

At birth (0 d), the percentage of apoptotic cells was significantly higher in the LE group than in the CON group, as determined by the detection of apoptosis using a terminal deoxynucleotidyl transferase dUTP nick end labeling (TUNEL) assay (Fig. 2G, P < 0.01). Proliferation was examined in testicular slices by immunohistochemical staining of PCNA (Proliferating Cell Nuclear Antigen) at 28 and 120 d; results revealed no significant difference in proliferation between the CON and LE groups (Fig. 3E, P > 0.05).

### Abundance and distribution of genes in boar testes based on RNA-seq

**Figure 4:**
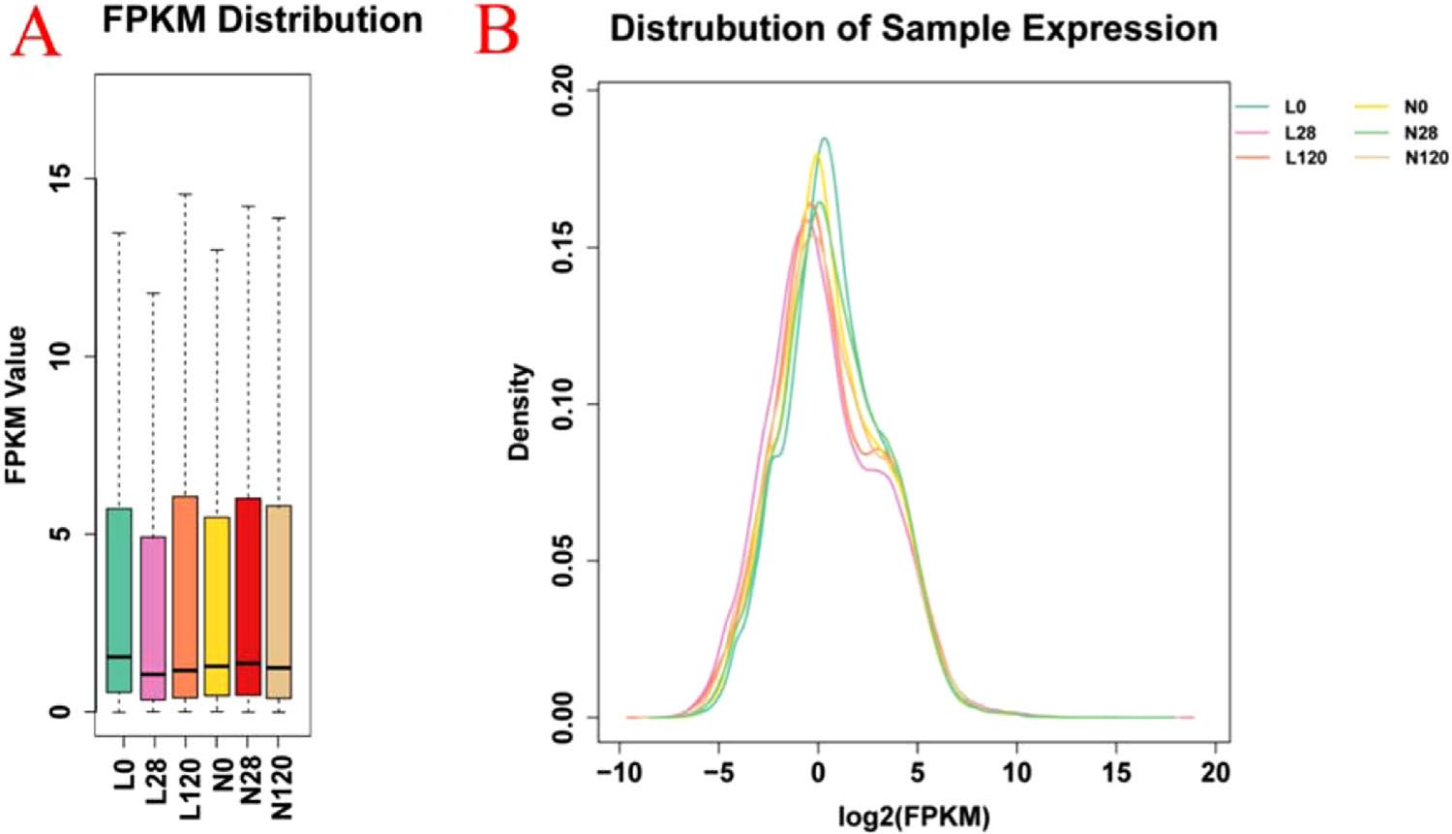
Abundant distribution of genes. (**A**) The FPKM value of each sample; (**B**) The gene expression distribution for each sample. FPKM = fragments per kilobase per million mapped reads.

In this study, six cDNA libraries (L0, L28, L120, N0, N28, and N120) were constructed using total RNA collected at 0, 28, and 120 d in the LE and CON groups. A total of 327,560,806 and 331,580,256 raw sequence reads were obtained through RNA-seq using RNA from the testes of the LE and CON groups, respectively (Table 2). After removal of the low-quality reads and adaptor sequences, 262,017,744 and 259,836,490 clean reads were selected for preparing the LE and CON group libraries, respectively. A total of 221,903,229 and 226,067,720 reads were mapped to the porcine reference genome of the LE and CON group boars, respectively (Table 2), and approximately 87% of the clean reads successfully aligned with the reference genome and the reference gene of pigs. Based on fragments per kilobase per million mapped reads (FPKM) analysis, the gene expression was classified into mRNA and lncRNA categories. A major proportion of genes was differentially expressed (−5 < log_2_FPKM < 5); most of the differentially expressed genes were expressed at moderate levels (log_2_FPKM ≥ 0). The results revealed a large proportion of genes with moderate differential expression (−5 < log_2_FPKM < 5) between the LE and CON boars (Fig. 4).

**Table 2:** Sample evaluation and sequencing data alignment.

Samples	L0	L28	L120	N0	N28	N120
Raw Reads Number	1.1E + 08	1.1E + 08	1.1E + 08	1.1E + 08	1.1E + 08	1.1E + 08
Raw Bases Number	1.7E + 10	1.6E + 10	1.6E + 10	1.7E + 10	1.7E + 10	1.6E + 10
Clean Reads Number	9.0E + 07	8.4E + 07	8.8E + 07	9.3E + 07	8.6E + 07	8.1E + 07
Clean Bases Number	1.4E + 10	1.3E + 10	1.3E + 10	1.4E + 10	1.3E + 10	1.2E + 10
Clean Reads Rate (%)	80.02	78.72	81.19	82.81	76.3	75.88
Raw Q30 Bases Rate (%)	89.36	87.13	88.4	90.52	86.39	86.11
Clean Q30 Bases Rate (%)	94.86	92.99	93.43	95.27	92.87	92.61
rRNA Reads Number	8.5E + 04	5.0E + 04	1.5E + 05	1.2E + 05	2.6E + 04	3.6E + 04
rRNA Mapping Rate (%)	0.09	0.06	0.17	0.13	0.03	0.04
Total Clean Reads Number	9.0E + 07	8.4E + 07	8.8E + 07	9.3E + 07	8.6E + 07	8.1E + 07
Total Clean Bases Number	1.4E + 10	1.3E + 10	1.3E + 10	1.4E + 10	1.3E + 10	1.2E + 10
Total Q30(%)	94.86	92.99	93.43	95.27	92.87	92.61
Total Reads	9.00E + 07	8.36E + 07	9.13E + 07	9.25E + 07	8.61E + 07	1.19E + 08
Mapped Reads	8.04E + 07	7.42E + 07	8.00E + 07	8.28E + 07	7.65E + 07	1.06E + 08
Mapping Rate	0.8928	0.8873	0.8765	0.8949	0.889	0.8934
Unmapped Reads	9.65E + 06	9.42E + 06	1.13E + 07	9.72E + 06	9.56E + 06	1.26E + 07
MultiMap Reads	6.58E + 06	5.38E + 06	6.09E + 06	6.68E + 06	5.84E + 06	8.44E + 06
MultiMap Rate	0.0731	0.0643	0.0667	0.0722	0.0679	0.0711

L0, L28, and L120 were newborn, 28 d, and 120 d, respectively, in the low energy (LE) group. N0, N28, and N120 were newborn, 28 d, and 120 d, respectively, in the control (CON) group.

Total Reads: the total number of sequences after filtering;

Mapped Reads: the number of sequences in the genome that are aligned;

Mapping Rate: the ratio of the number of sequences in the genome that are aligned;

Unmapped Reads: the number of sequences in the genome that are not aligned;

MultiMap Reads: the number of sequences aligned to multiple locations in the genome;

MultiMap Rate: the ratio of the number of sequences aligned to multiple locations in the genome.

### DEGs between LE and CON boar testes

**Figure 5:**
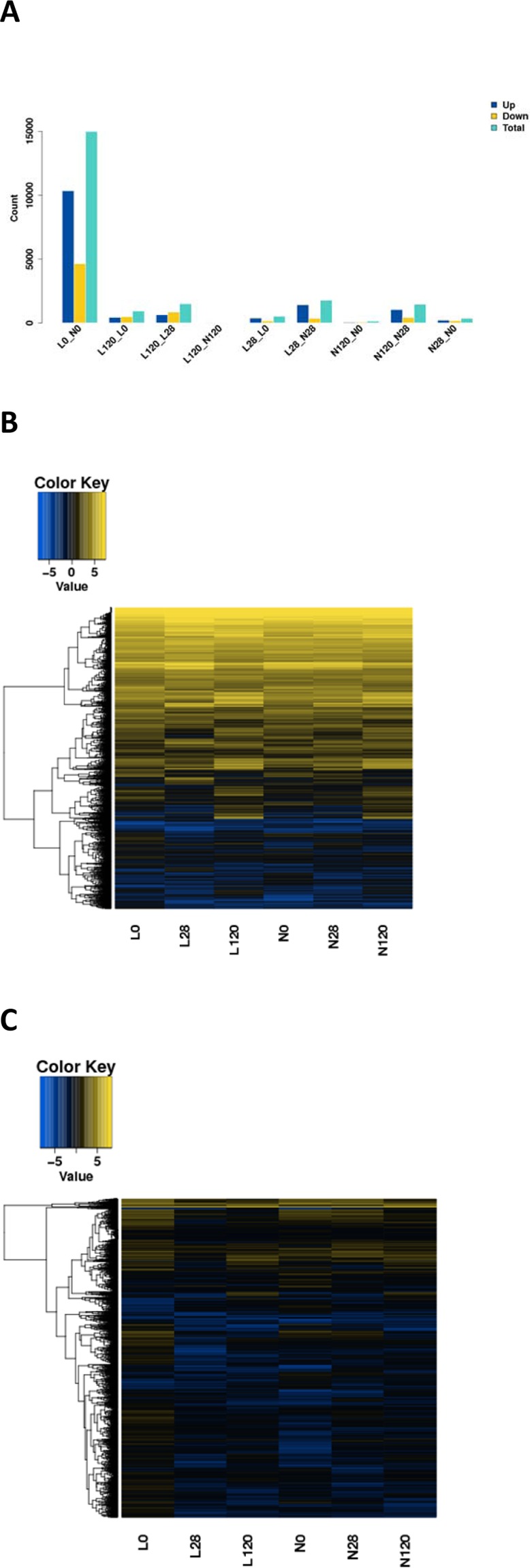
Analysis of differentially expressed genes (DEGs). (**A**) the number of differential gene distribution in the testis (|log2Ratio| ≥ 1, q < 0.05). (**B**,**C**) The heatmap of the DEG subsets (|log2Ratio| ≥ 1, q < 0.05) in different samples (**B**, mRNA; **C**, lncRNA).

After the differential expression analysis, transcriptome sequencing results showed that the numbers of DEGs in the comparison groups L0-N0, L28-N28, and L120-N120 (L represents LE, and N represents CON) were 14988, 1772, and 25, respectively; among them, the mRNA numbers were 6066, 1396, and 19, and the numbers of lncRNAs were 8922, 376, and 6 (|log_2_ratio| ≥ 1, q < 0.05), respectively, as presented in Fig. 5A.

Next, we filtered significant DEGs (|log_2_ratio| ≥ 1, q < 0.05) between the groups at the three developmental stages (0, 28, and 120 d). The heatmap (Fig. 5B,C) revealed two main sample branches (L0, L28, and L120 versus N0, N28, and N120). This finding suggested that the expression patterns of the two groups at 0, 28, and 120 d were different, and significant DEGs between the two groups at time points 0 and 28 d were substantially different from those at the 120 d time point.

### Gene ontology (GO) and kyoto encyclopedia of genes and genomes (KEGG) pathway analyses of DEGs with respect to testicular development

The differentially expressed mRNAs and lncRNAs were classified according to GO categories and were found to be mainly associated with the GO terms, “cellular process,” “metabolic process,” “cell,” “cell part,” “binding,” “membrane part,” and “signal transduction (signal transducer)” (Supplementary Fig. 1). The results revealed that for the differentially expressed mRNAs, the most enriched GO categories were spread across 405 biological processes, 94 cellular components, and 155 molecular functions. Moreover, the results revealed that for the differentially expressed lncRNAs, the most enriched GO categories were spread across 698 biological processes, 271 cellular components, and 193 molecular functions.

**Figure 6:**
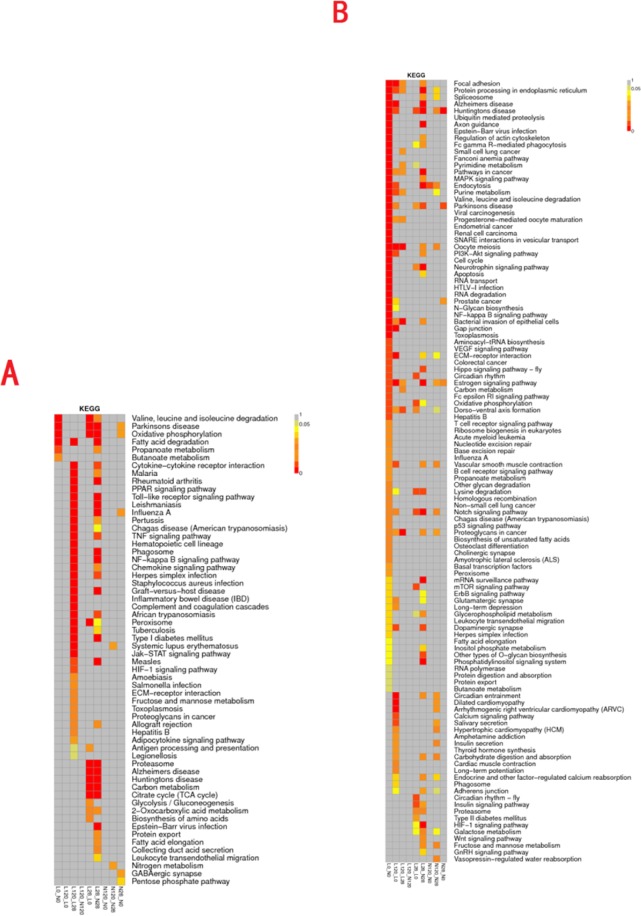
KEGG enrichment map of differentially expressed genes. KEGG enrichment map of mRNA (**A**) and lncRNA (**B**). The ordinate is the name of the KEGG metabolic pathway, and the abscissa is the name of the two comparison groups.

As depicted in the KEGG enrichment map of mRNAs (Fig. 6A), fewer pathways that correlated with testicular development were found in the L0-N0 comparison group. In the L28-N28 comparison group, enriched pathways (P < 0.05) that were significantly associated with testicular development included fatty acid degradation and apoptosis pathways. As illustrated in the KEGG enrichment map of lncRNAs (Fig. 6B), the most significantly enriched pathways in the L0-N0 comparison group were the MAPK, cell cycle, PI3K-AKT, and oxidative phosphorylation signaling pathways. In the L28-N28 comparison group, the significantly enriched pathways included the PI3K-AKT signaling pathway. In this study, eight important pathways related to the regulation of male sexual function were identified (Table 3), which indicated a large difference in testicular development between these groups. The eight pathways included the largest number of DEGs that were detected in the comparison groups, L0-N0 and L28-N28, thus indicating large differences in testicular development at these time points.

**Table 3:** The number of all genes and differentially expressed genes (DEGs) annotated in the pathways.

*Pathway	DEGs with pathway annotation in mRNA			DEGs with pathway annotation in lncRNA		
	L0 vs N0	L28 vs N28	L120 vs N120	L0 vs N0	L28 vs N28	L120 vs N120
Fatty acid degradation	27	13	—	—	—	—
Cell cycle	—	—	—	39	—	—
Apoptosis	—	—	—	26	9	—
Pi3k-AKT signaling pathway	—	—	—	105	25	—
mTOR signaling pathway	—	—	—	15	—	—
P53 signaling pathway	—	—	—	22	—	—
MAPK signaling pathway	—	—	—	86	14	—
Oxidative phosphorylation	65	45	—	60	34	—

*The eight pathways are related to boar sexual function.

### Verification of DEGs by quantitative reverse-transcription PCR (qRT-PCR)

**Figure 7:**
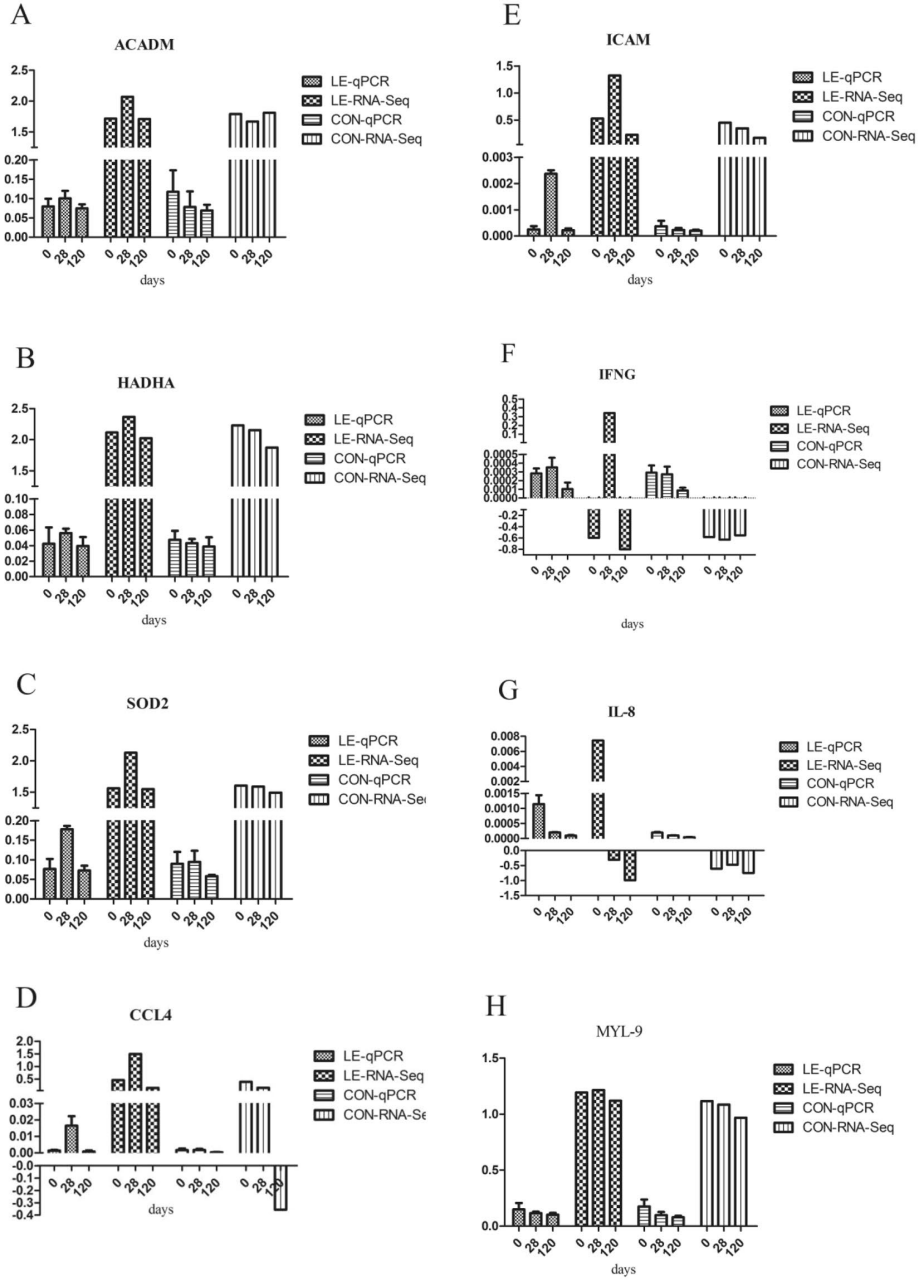
Verification of differential genes by RT-PCR. Expression levels of eight mRNAs (A-H) were measured by qRT-PCR, named LE-qPCR and CON-qPCR. The mRNA levels were expressed as means ± standard deviation of three biological replicates. The clean reads of each mRNA molecule obtained by HiSeq technology were also standard by the bar of the expression levels by the Y-axis on a log10 scale, named LE-RNA-seq and CON- RNA-seq.

To evaluate our DEG library, the expression of eight DEGs was analyzed by qRT-PCR. The results of the qRT-PCR revealed the same expression pattern that was observed in the RNA-seq analysis (Fig. 7), thereby confirming the reliability of our data.

### Quantification of the mTOR, AMPK, and S6 proteins

**Figure 8:**
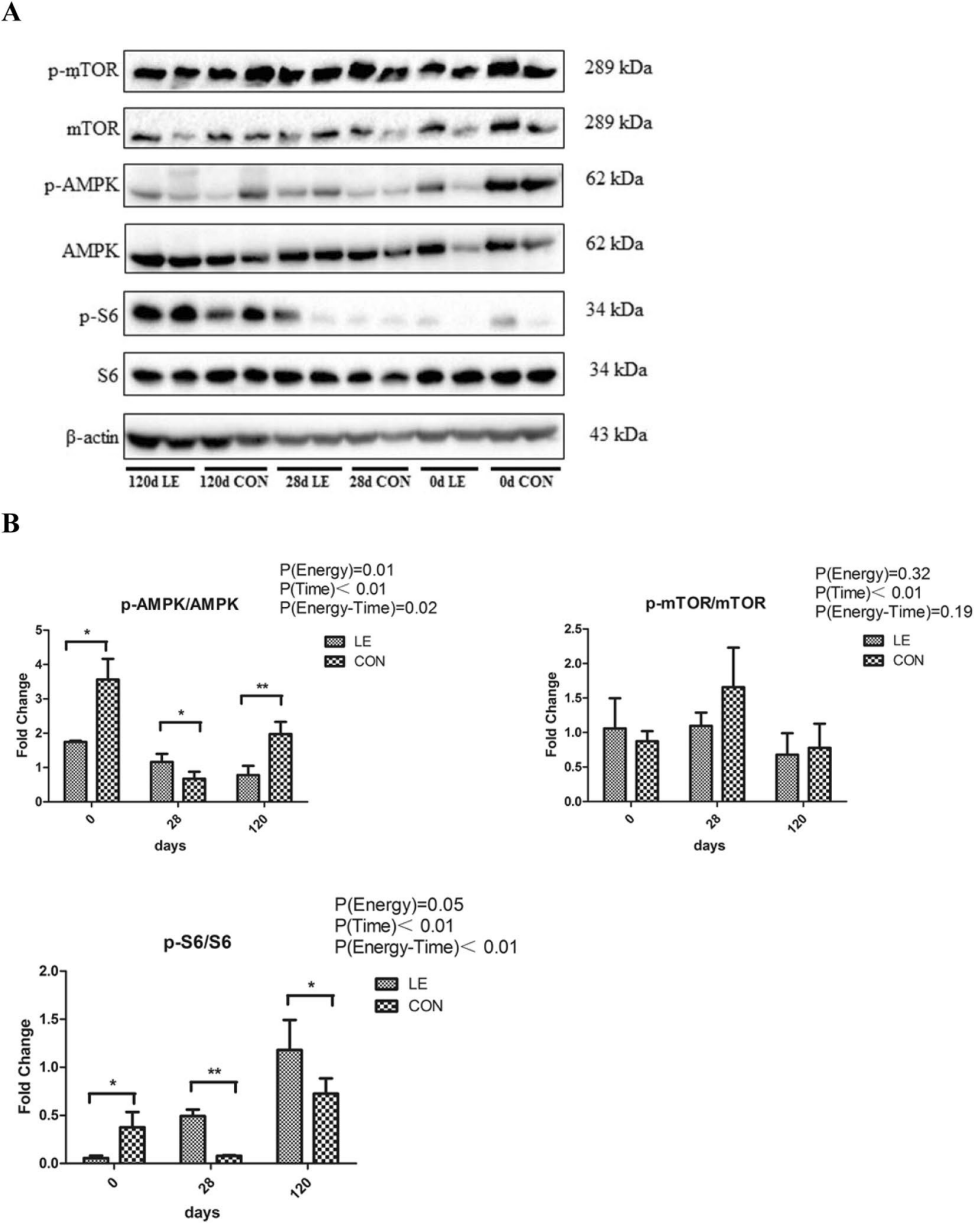
Effect of maternal energy restriction during gestation on testis protein expression in boar offspring. *P < 0.05, **P < 0.01.

Based on the transcriptome analysis, mTOR, AMPK, and S6 were selected for protein expression analysis. The results revealed that the phospho- (p-)AMPK/AMPK ratio was significantly lower in the LE group than in the CON group at 0 d and 120 d (P < 0.05), and was significantly higher than that in the CON group at 28 d. The p-S6/S6 ratio in the LE group was significantly lower than that in the CON group at 0 d and significantly higher than that in the CON group at 28 and 120 d (P < 0.05). The p-mTOR/mTOR ratios were not significantly different between groups (Fig. 8).

## Discussion

These results revealed that restricting maternal energy intake during pregnancy reduces the testicular weight at birth and the number of germ cells in newborn boars, and that this effect lasts up to 120 d and is likely to continue into adulthood. These findings imply that insufficient maternal energy has a negative influence on offspring testicular development. Some studies indicate that low energy intake during gestation decreases testicular weight and cell numbers^13,14^ in the offspring; studies on the limitation of other nutritional parameters have yielded similar results^18,19^. In addition, we found that seminiferous tubule diameters and testicular cell numbers were larger at 120 d after birth than at 28 d post-birth, suggesting that a rapid proliferation of Sertoli cells took place between 28 and 120 d. The proliferation and apoptosis of germ cells are important mechanisms during testicular development and spermatogenesis, as is the blood–testis barrier. In this study, low maternal energy intake had no effect on cell proliferation; however, it increased testicular apoptosis and upregulated apoptosis-related genes (Supplemental Table 1); these data may explain the decrease in cell numbers.

The transcriptome data generated in this study will be helpful for elucidating the mechanism by which maternal nutrition affects testicular development and the critical period of this effect. It has been reported that more than 18 million reads from RNA-seq analyses are required for each sample to attain a saturation point of novel gene discovery and expressional analysis^20,21^. Our quality control procedures (including analysis of base sequence quality and distribution) revealed that our RNA-seq data were well qualified for such analyses (Table 2). An interesting finding in the DEG data was that lncRNAs and mRNAs were mainly concentrated in the offspring testis comparison groups at 0 and 28 d, suggesting that the maternal effect mainly occurs during the early stages of life. In mammals including humans, testicular development mainly takes place at the early stages of life^22–24^, in boars, the number of Leydig and Sertoli cells increases by approximately 6-fold between birth and 1 month of age^24^. Some studies also suggest that a low maternal energy level can reduce the testicular weight and the number of Sertoli cells in newborn males^13^, thereby possibly exerting a negative impact on subsequent fertility in adulthood. Therefore, we can get some clues from the finding that the expression of lncRNAs and mRNAs that are mainly involved in the regulation of testicular development early in life was affected by maternal energy restriction.

The number of lncRNAs in the DEGs was significantly higher than that of mRNAs. To our knowledge, this is the first study to show that lncRNAs are involved in mediating the effect of maternal energy insufficiency on testicular development in the offspring. Some reports indicate that lncRNAs regulate development and the maintenance and differentiation of stem cells^25,26^. The expression of lncRNAs increases significantly when sperm cells enter the meiotic phase, and this growth trend persists from spermatogonial differentiation to the round sperm cell stage^27,28^. Sun^29,30^ found that lncRNAs are abundant in the testes, and that the overall number of lncRNAs changed in expression are similar to that of mRNAs. In mammals, lncRNAs play an important role in testicular development and spermatogenesis^31^, and are involved in sex determination, gonadal development, and mediating the sex hormone response^32^. Thus, we can speculate that lncRNAs may have a greater role in testicular development than mRNAs.

GO is a bioinformatic database that is widely used to study functional relations between genes. We identified several major GO terms in our GO analysis of all differentially expressed mRNAs and lncRNAs, and the results implied that these genes are related to testicular development.

Pathway analysis of significant DEGs, based on the KEGG database, clustered the DEGs into eight pathways that regulate boar testicular development. We detected a large number of DEGs belonging to these pathways between the LE and CON groups at different ages (Table 3), suggesting that most of these DEGs encode lncRNAs. KEGG analysis showed that the PI3K-AKT, mTOR, cell cycle, and cell apoptosis signaling pathways are involved in the regulation of testicular development. Tanwar found that the AMPK–PI3K–mTOR signaling pathway plays an important role in Sertoli cell and testicular development^33^. AMPK is a crucial cellular energy sensor protein that is activated by a low energy state in the cell^34^, whereas mammalian target of rapamycin (mTOR) is one of the downstream targets of AMPK and functions as an intracellular nutrient sensor to control cell growth and metabolism^35^. The AMPK–mTOR axis has been implicated as a mediator between gonadal function and energy balance. In this study, maternal nutrition and developmental stages had different effects on mRNA expression of *AMPK*, *mTOR*, *S6K*, *MAP2K1*, *PI3K*, *4EBP*, and *AKT* (Supplemental Table 1). The protein amounts of AMPK, p-AMPK, S6, and p-S6 in offspring testes were affected by prenatal energy levels, developmental stages, and their interactions, whereas the expression of mTOR and p-mTOR proteins in the testes of the offspring was affected by developmental stages, but was unaffected by maternal nutrition. These findings indicate that the AMPK–mTOR pathway is important for mediating the effect of low maternal energy intake in such a manner that it effects testicular development. In fact, AMPK is also localized in mouse Sertoli cells^36^, and specific deletion of the *AMPKα1* gene in the Sertoli cells of mice has been shown to lead to a 25% reduction in male fertility that is associated with abnormal spermatozoa^36^. It is generally known that the mTOR pathway plays an important role in the regulation of meiotic progression of male germ cells in mice^37^, proliferation of rat Sertoli cells^38^, and DBP-induced apoptosis of testicular Sertoli cells^39^. Inhibition of mTOR protein expression with rapamycin has been found to impact the proliferation and differentiation of spermatogonial cells in the testicular tissue, by reducing the amounts of p-p70S6K and p-4EBP1 and by downregulating a key meiosis initiation gene (*STRA8*)^40^, thereby blocking the proliferation of spermatogonia and significantly decreasing the number of spermatozoa in SD mice^41^. mTOR phosphorylates and modulates the activity of the serine/threonine ribosomal protein S6 kinase B1 (S6K1). In turn, S6K1 phosphorylates and activates S6, a ribosomal protein involved in translation^42^. These findings suggest that the AMPK–mTOR–p70S6K–4EBP1 pathway may be involved in the regulation of testicular development and cell number in response to low maternal energy intake during pregnancy.

Another interesting phenomenon uncovered in this study is the involvement of known lncRNAs (such as those involved in the MAPK signaling pathway) in the regulation of testicular development during low maternal energy intake. To date, no studies have indicated whether MAPK is involved in the programming effect of maternal energy on testis development in the offspring. It has been reported that MAPK signaling pathways can regulate mammalian reproduction^43^, cell proliferation, and spermatogenesis^44^. In addition, the MAPK–extracellular regulated protein kinase 1 and 2 (ERK1/2) pathway is involved in the regulation of intercellular events that control the formation and maintenance of the blood–epididymis barrier^45^. There is evidence that vitamins E and C improve spermatogenesis and maintain the blood–testis barrier, mainly through the p38 MAPK signaling cascade^46,47^. Another important function of MAPK is the regulation of the inflammatory and innate immune responses in the testes^48^. Local testicular immune homeostasis is important for male fertility. Zhang^49^ found that the lncRNA set is mainly enriched in MAPK signaling pathways in sheep testes and changes with dietary energy. We concluded that the change in expression of these lncRNAs may be specific and might be crucial for testicular development in the offspring; these findings require further confirmation and research.

In summary, maternal energy intake is involved in the programming of offspring testicular development. Maternal energy intake modulates the cell number and tissue structure in the testes of boar offspring at an early stage of life. Additionally, lncRNA-, as well as mRNA-mediated regulation may affect spermatogenesis in adult boars. These data will provide a valuable transcriptomic resource for modelling the effects of maternal energy intake on offspring testis development and would enable researchers to identify functional genes, molecular markers, and pathways that influence testicular development and spermatogenesis.

## Materials and Methods

The study protocol was reviewed and approved by the Institutional Animal Care and Use Committee of Sichuan Agricultural University (Ya’an, Sichuan, China) and complied with the current laws on animal protection (Ethical Approval Code: SCAUAC201408-3)^4^.

### The animals and sample collection

A total of 36 LY (Landrace × Yorkshire) sows (7–9 parity) with similar backfat were selected from a research farm at the Sichuan Agricultural University. During pregnancy, the sows were randomly assigned to one of the following diets: a diet containing 3.40 Mcal of digestible energy per kilogram (CON group) or a low-energy diet containing 3.00 Mcal of digestible energy per kilogram (LE group). The sows were fed these diets at 2.2 kg/d during early pregnancy (1–30 d of gestation), 2.4 kg/d during mid-pregnancy (31–90 d), and 2.8 kg/d during late pregnancy (91 d to parturition). After farrowing, all the sows were fed the same diet in accordance with NRC 2012^4^. A total of 15 healthy offspring boars were selected from the CON or LE groups, and both groups were fed the same basic boar diet (Ref. NRC2012) until 120 d.

Testes of offspring boars were collected at 0, 28, and 120 d after birth from different litters by castration, parts of the testes were frozen for RNA extraction. Others were fixed in a 4% formaldehyde solution for histological and morphological analyses.

### Light microscopy

The methods of making slices were based on the protocol proposed by Ding^50^. First, five testicular samples of different ages per group were fixed in 4% formaldehyde solution. Each sample was dehydrated, cleared, and sectioned. Then, the cross-section was stained with HE, and photographed using Nikon Eclipse TS100 biomicroscope. Only round seminiferous tubule sections were selected for counting. The diameters of each cross-section were measured using the Image Pro-Plus software at 200 × magnification in 10 randomly selected visual fields. The numbers of Leydig^51,52^, germ, and Sertoli cells^13,53^ were also determined in 10 visual fields at 200 × magnification at each cross-section. Daily sperm production counts were estimated by a histometric method based on round spermatids^54,55^.

### Immunohistochemical analysis

Testes sections from the LE and CON groups were deparaffinized with xylene and ethanol solutions. The slices were recovered with EDTA-containing antigen retrieval buffer (pH 9.0) and blocked for 25 min with a 3% hydrogen peroxide solution (hydrogen peroxide: pure water at 1:9, v/v). Subsequently, the slices were blocked with 3% BSA or 10% rabbit serum, and incubated for 12 h with a primary antibody: anti-PCNA (D120014; Shanghai, Sangon Biotech). Next, the sections were washed with PBS and incubated with a secondary antibody (D120014; Shanghai, Sangon Biotech) for 50 min. The slides were cleaned three times with PBS (pH7.4) for 5 minutes (per wash). After drying the slices, a freshly prepared DAB coloring solution was added dropwise, and the brown-yellow stained slides were then counterstained with hematoxylin and photographed under a microscope (Nikon Eclipse TI-SR).

### TUNEL assay

First, the paraffin sections were deparaffinized with xylene and ethanol, respectively, pretreated with a proteinase K working solution (proteinase K stock solution diluted with PBS 1:9), and incubated with TdT and dUTP (TdT: dUTP = 2:29; Roche, 11684817910) for 2 h at 37 °C. Then, endogenous peroxidase in the slides was blocked with a 3% hydrogen peroxide solution (hydrogen peroxide: methanol = 1:9, v/v), and the slides were covered in converter-POD (Roche, 11684817910) and incubated at 37 °C for 30 min. After the slides were washed with PBS, freshly prepared DAB (DAKO, K5007) coloring solution was added dropwise, and slides with brown-yellow staining were then counterstained with hematoxylin and photographed under the microscope (Nikon Eclipse TI-SR).

### Library preparation and quantification

RNA-seq was performed by Annoroad Gene Technology Co., Ltd. (Beijing, China). The RNA-seq methods were as described by Shi^56^. Total RNA was extracted from the testes collected at 0, 28, and 120 d from boars born in the CON group (designated N0, N28, N120) or LE group (designated L0, L28, and L120) using the TRIzol reagent (Invitrogen). Sequencing libraries were generated using the NEBNext® Ultra™ RNA Library Prep Kit for Illumina® (#E7530L, NEB, USA)^56^.

### Mapping and DEG analysis

The reference genomes were derived from the ENSEMBL database (http://www.ensembl.org/index.html), and clean data were mapped to the reference genome using HISAT2 (http://ccb.jhu.edu/software/hisat2/index.shtml)^56^. FPKM values were used to calculate the expression of genes in each sample. Differential expression was analyzed by the DEGseq software (http://www.bioconductor.org/packages/release/bioc/html/DEGseq.html), Genes with q ≤ 0.05 and |log_2_ratio| ≥ 1 were considered as DEGs^56^.

### GO and KEGG pathway enrichment analyses

GO (http://geneontology.org/) enrichment analysis revealed the biological functions of the DEGs. GO terms with q < 0.05 were considered significantly enriched. For the functional classification of genes, by the same method as in the GO analysis, significantly enriched KEGG (http://www.kegg.jp/) pathways were identified^56^.

### qRT-PCR validation of the DEGs

Changes in the expression of eight randomly selected genes were validated by qRT-PCR (TaKaRa, Dalian, China)^50^, the expression of 22 testicular-development–related genes was also quantified. All qRT-PCR experiments were performed in triplicate on an Applied Biosystems 7900HT Real-Time PCR Detection System. The primers are listed in Supplementary Table 2. The endogenous control genes for β-actin, were used in these experiments. The 2^−ΔΔCt^ method was used to analyze the relative gene expression^57^.

### Western blotting

Western blotting was carried out as described by Feng and Hua^58,59^. The reagents employed during this assay were as follows: lysis buffer (cat. # P0013B; Beyotime Institute of Biotechnology, Jiangsu, China); protease inhibitor cocktail (04693132001; Roche, Basel, Switzerland); BCA Protein Assay Kit (23227; Thermo Fisher Scientific); polyvinylidene fluoride membrane (1620177; Bio-Rad Laboratories, Hercules, CA, USA); antibodies against β-actin (4970S), p-mTOR (2971S), mTOR (2972S), p-S6 (4858 T), ribosomal protein S6 (S6; 2217S), p-AMPK (2535S), and AMPK (2793S); and secondary antibodies (7074 and 7076; Cell Signaling Technology). Protein signals were detected with the ECL western blotting detection reagent (1705060; Bio-Rad Laboratories) on a Molecular Imager Chemi Doc XRS+ System (Bio-Rad Laboratories)^59^. The signals on the blots were quantified using the Image J software (National Institutes of Health, Bethesda, MD, USA)^59^.

### Statistical analyses

Statistical analyses were performed for the testicular weight and histological data of all the boars. Data were analyzed via unpaired Student’s two-tailed *t* test. The other data (immunohistochemistry, TUNEL assay, and western blotting) were analyzed as a 2 × 2 factorial with the general linear model procedures of the Statistical Analysis Package^60^. The model factors included the effects of energy, time, and their interaction. All data were analyzed in the SAS software (Version 9.2; SAS Institute Inc., Cary, NC, USA) and reported as means with their SDs. Data with P < 0.05 were considered statistically significant, whereas data with P < 0.10 were considered to be a tendency.

## Supplementary information

Supplemental Figure 1

Supplemental Table 1

Supplemental Table 2
