# Supplementary material for: Maternal energy insufficiency affects testicular development of the offspring in a swine model
Source: Sci Rep. 2019 Oct 10;9:14533. doi: 10.1038/s41598-019-51041-y (PMC6787339; doi:10.1038/s41598-019-51041-y)
Supplement: Supplementary file 1 — Supplemental Figure 1 [file 41598_2019_51041_MOESM1_ESM.pdf]

Maternal energy insufficiency affects testicular development of the offspring in a swine model

Yan Lin[#*1], Xue-Yu Xu[#1], De Wu[1], Hao Lin[2], Zheng-Feng Fang[1], Bin Feng[1], Sheng-Yu Xu[1], Lian-Qiang Che[1], Jian Li[1], Yong Zhuo[1], Cai-Mei Wu[1], Jun-Jie Zhang[3], Hong-Jun Dong[1]

[1]Key Laboratory for Animal Disease Resistance Nutrition of the Ministry of Education of China, Institute of Animal Nutrition, Sichuan Agricultural University, Chengdu, Sichuan, China. 611130.

[2]Key laboratory for Neuro-Information of Ministry of Education, School of Life Science and Technology, Center for Informational Biology, University of Electronic Science and Technology of China, Chengdu 610054

[3]School of Life Science, Sichuan Agricultural University, Ya'an, Sichuan, China. 625014.

# Authors have contributed equally to this work.

* Corresponding author. Tel.: +086 835 2885065; Fax: +086 835 2885065.

E-mail address: linyan936@163.com

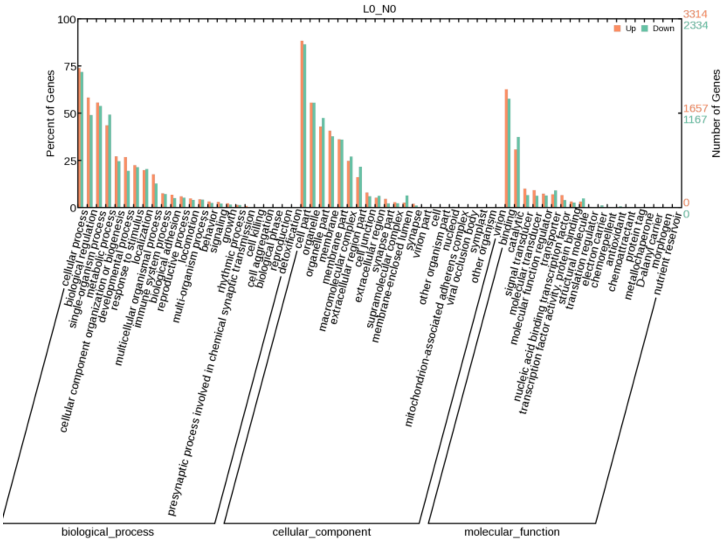

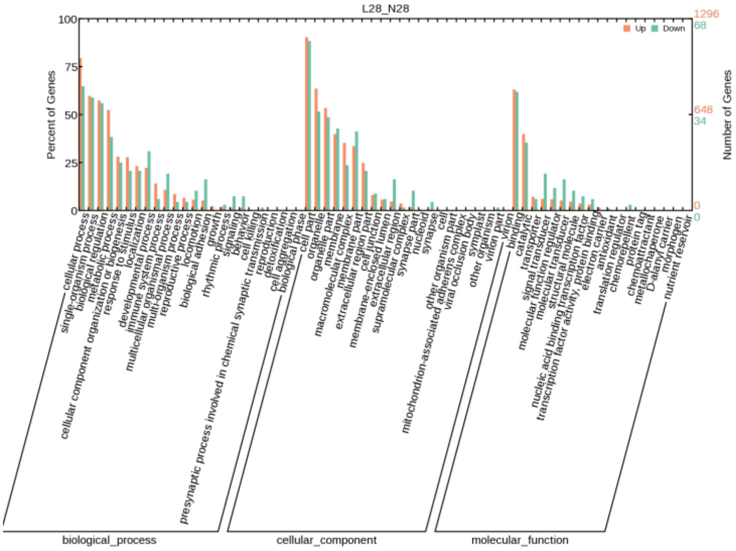

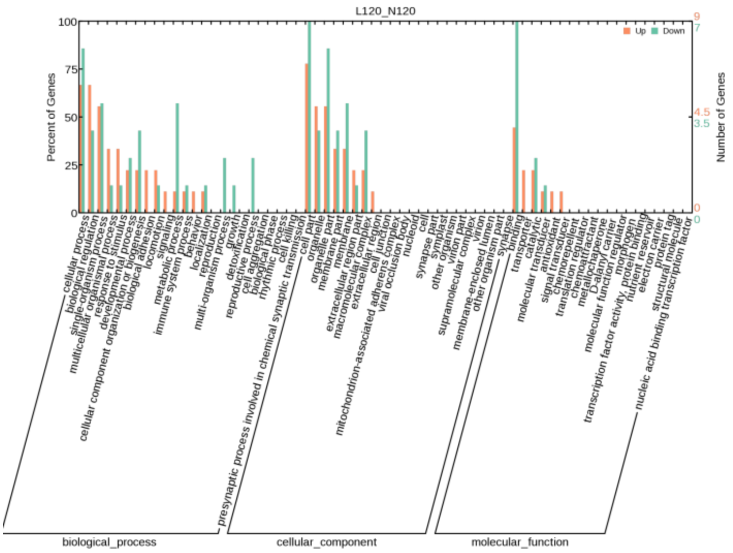

Supplemental Figure 1: The GO map of comparison group at each timepoint. Gene ontology (GO) classification of the differentially expressed genes in the testis of offspring boar from control diet(N) sows or low-energy diet(L) sows. The numbers represent the number of differentially expressed genes associated with a given GO term. L0, L28, and L120 were newborn, 28 days, and 120 days in low energy (LE) group, respectively. N0, N28, and N120 were newborn, 28 days, and 120 days in control days (CON) group, respectively.
