# Supplementary material for: Maternal energy insufficiency affects testicular development of the offspring in a swine model
Source: Sci Rep. 2019 Oct 10;9:14533. doi: 10.1038/s41598-019-51041-y (PMC6787339; doi:10.1038/s41598-019-51041-y)
Supplement: Supplementary file 2 — Supplemental Table 1 [file 41598_2019_51041_MOESM2_ESM.pdf]

Maternal energy insufficiency affects testicular development of the offspring in a swine model

Yan Lin[#*1], Xue-Yu Xu[#1], De Wu[1], Hao Lin[2], Zheng-Feng Fang[1], Bin Feng[1], Sheng-Yu Xu[1], Lian-Qiang Che[1], Jian Li[1], Yong Zhuo[1], Cai-Mei Wu[1], Jun-Jie Zhang[3], Hong-Jun Dong[1]

[1]Key Laboratory for Animal Disease Resistance Nutrition of the Ministry of Education of China, Institute of Animal Nutrition, Sichuan Agricultural University, Chengdu, Sichuan, China. 611130.

[2]Key laboratory for Neuro-Information of Ministry of Education, School of Life Science and Technology, Center for Informational Biology, University of Electronic Science and Technology of China, Chengdu 610054

[3]School of Life Science, Sichuan Agricultural University, Ya'an, Sichuan, China. 625014.

# Authors have contributed equally to this work.

* Corresponding author. Tel.: +086 835 2885065; Fax: +086 835 2885065.

E-mail address: linyan936@163.com

Item	Time	LE	CON	*P*-value		
				Energy	Time	Energy-time
ACADM	0d	0.63±0.11[b]	1.00±0.19[a]	<0.01	<0.01	0.15
	28d	0.51±0.13	0.67±0.07			
	120d	0.42±0.03	0.55±0.01			
HADHA	0d	0.62±0.20[b]	1.00±0.04[a]	0.06	0.02	<0.01
	28d	1.13±0.07	0.85±0.10			
	120d	0.59±0.02[b]	0.86±0.10[a]			
P450scc	0d	0.07±0.00[b]	1.00±0.01[a]	0.51	<0.01	<0.01
	28d	3.73±0.27[a]	2.92±0.19[b]			
	120d	1.59±0.39	1.16±0.62			
CYP19A	0d	2.23±0.12[a]	1.00±0.35[b]	<0.01	<0.01	<0.01
	28d	1.04±0.08	0.96±0.12			
	120d	20.77±1.54[b]	50.38±10.77[a]			
GATA4	0d	0.93±0.12	1.00±0.27	0.14	<0.01	0.68
	28d	0.77±0.18	0.97±0.10			
	120d	0.50±0.03	0.56±0.01			
STRA8	0d	0.80±0.10	1±0.20	0.36	<0.01	0.69
	28d	0.50±0.10[b]	1.30±0.20[a]			
	120d	6.00±2.20	9.50±2.10			
PCNA	0d	1.58±0.38	1.00±0.15	0.11	<0.01	0.02
	28d	1.17±0.05	0.99±0.17			
	120d	1.88±0.21	2.10±0.26			
CCND2	0d	1.38±0.02[a]	1.00±0.06[b]	0.16	<0.01	<0.01
	28d	0.56±0.03[b]	1.14±0.17[a]			
	120d	0.18±0.01[a]	0.13±0.02[b]			
GADD45	0d	1.21±0.40	1.00±0.14	0.85	<0.01	0.28
	28d	0.64±0.01	0.65±0.13			
	120d	0.87±0.13	1.01±0.07			

CCNA	0d	1.09±0.10	1.00±0.21	0.48	<0.01	0.91
	28d	0.62±0.07	0.58±0.08			
	120d	0.91±0.21	0.89±0.16			
C-kit	0d	0.96±0.08	1.00±0.20	0.23	<0.01	0.81
	28d	0.39±0.06	0.50±0.08			
	120d	0.28±0.06	0.33±0.08			
CYC	0d	0.86±0.11	1.00±0.12	<0.01	<0.01	0.45
	28d	0.34±0.09[b]	0.46±0.02[a]			
	120d	0.50±0.12[b]	0.76±0.09[a]			
Caspase3	0d	1.20±0.20	1.00±0.12	0.37	<0.01	0.16
	28d	0.92±0.06	1.01±0.22			
	120d	0.32±0.04	0.28±0.01			
CFLAR	0d	1.06±0.15	1.00±0.07	0.36	<0.01	0.21
	28d	0.85±0.13	1.09±0.30			
	120d	0.35±0.03	0.36±0.01			
Caspase10	0d	0.95±0.15	1.00±0.05	0.19	<0.01	<0.01
	28d	1.40±0.10[b]	1.75±0.15[a]			
	120d	1.05±0.05[a]	0.85±0.15[b]			
MAP2K1	0d	0.76±0.08	1±0.25	0.87	<0.01	0.17
	28d	1.18±0.27	1±0.24			
	120d	0.81±0.32	0.65±0.10			
PI3K	0d	1.31±0.45	1.00±0.38	0.31	<0.01	0.6
	28d	1.23±0.08	1.00±0.08			
	120d	0.35±0.04	0.38±0.04			
AMPK	0d	1.77±0.30[a]	1.00±0.11[b]	0.39	<0.01	<0.01
	28d	0.73±0.15	0.92±0.15			
	120d	0.55±0.07[b]	0.87±0.32[a]			
mTOR	0d	1.36±0.06	1.00±0.13	0.83	<0.01	0.13
	28d	0.78±0.16	0.75±0.03			
	120d	1.26±0.18	1.57±0.57			
S6K	0d	1.67±0.38[a]	1.00±0.13[b]	0.03	<0.01	0.01
	28d	0.78±0.17	0.84±0.07			
	120d	0.59±0.11	0.55±0.04			
4EBP	0d	1.13±0.18	1.00±0.20	0.08	<0.01	0.48
	28d	0.80±0.04	0.61±0.10			
	120d	0.64±0.11	0.63±0.03			
AKT	0d	0.96±0.26	1.00±0.14	<0.01	<0.01	<0.01
	28d	0.55±0.12	0.69±0.18			
	120d	0.71±0.11[b]	1.92±0.26[a]			

Supplemental Table 1: The related gene expression of testicular. (Mean ±SD);
a, b Means in rows with different superscript litters are different（P<0.05）.
