# Supplementary material for: Maternal energy insufficiency affects testicular development of the offspring in a swine model
Source: Sci Rep. 2019 Oct 10;9:14533. doi: 10.1038/s41598-019-51041-y (PMC6787339; doi:10.1038/s41598-019-51041-y)
Supplement: Supplementary file 3 — Supplemental Table 2 [file 41598_2019_51041_MOESM3_ESM.pdf]

Maternal energy insufficiency affects testicular development of the offspring in a swine model

Yan Lin[#*1], Xue-Yu Xu[#1], De Wu[1], Hao Lin[2], Zheng-Feng Fang[1], Bin Feng[1], Sheng-Yu Xu[1], Lian-Qiang Che[1], Jian Li[1], Yong Zhuo[1], Cai-Mei Wu[1], Jun-Jie Zhang[3], Hong-Jun Dong[1]

[1]Key Laboratory for Animal Disease Resistance Nutrition of the Ministry of Education of China, Institute of Animal Nutrition, Sichuan Agricultural University, Chengdu, Sichuan, China. 611130.

[2]Key laboratory for Neuro-Information of Ministry of Education, School of Life Science and Technology, Center for Informational Biology, University of Electronic Science and Technology of China, Chengdu 610054

[3]School of Life Science, Sichuan Agricultural University, Ya'an, Sichuan, China. 625014.

# Authors have contributed equally to this work.

* Corresponding author. Tel.: +086 835 2885065; Fax: +086 835 2885065.

E-mail address: linyan936@163.com

Primer name	Primer sequence（5'-3'）	Login number
mTOR-R	CCT TTC GAG ATG GCA ATG GA	GU086214.1
mTOR-F	TTG TTG CCC CCT ATT GTG AAG	
P70S6K-R	TTG GAA GTG GTG CAG AAG CTT	XM_003131671.4
P70S6K-F	GGA AAC AAG TGG AAT AGA GCA GAT G	
PI3K-R	AAC CAC GCT TCA GCA GAA AT	Y10743.1
PI3K-F	CCA GAA ACT GGA GAG CTT GG	
AMPK-R	AGC TCG CCT CCT GAA ACA TA	NM_001167633.1
AMPK-F	CCG CAG AGA AAT TCA GAA CC	
STAR8-R	AAG GTC CAT CGT CTG CTT GT	JQ965783.1
STAR8-F	GAG GAG GAG GAA GAG GAG GA	
GATA4-R	TGA TGC CAT TCA TCT TGT GG	AY115491.1
GATA4-F	TCT CGG AAG GCA GAG AGT GT	
CDK1-R	TTT CGA GAG CAG ATC CAA GC	NM_001159304.2
CDK1-F	AGT GTG GCC AGA AGT GGA GT	
Cyclinb1-R	TGC TTT GTA AGC CCT CGA TT	NM_001170768.1
Cyclinb1-F	TGG TGA ATG GAC ACC AAC TC	
P450SCC-R	AAT GCT GGT GAT AGG CAA CC	L34259.1
P450SCC-F	TCG GCA ACT TGG AAT CTG TT	
FasL-R	CCC GGA AGT ACA CTT TGG AA	AY033634.1
FasL-F	CTC TGG AAT GGG AAG ACA CC	
C-kit-R	TTC CAC GAG GTC ATC AAC AA	L07786.1
C-kit-F	AAG CGA AAT GGT GGA ACA AC	
AKT-F	GAG GTC ATG GAG CAC AGG TT	NM_001256779.1
AKT-R	CGT CAA AGT ACC GAG TGT CG	
CYP19A-F	TGG AGT GCA TCG GCA TGT AT	NM_214430.1
CYP19A-R	GTG ATG GAA TCG GCA CAG AC	
CYC-F	GGC TCC TGG ATT CTC TTA CAC AG	NM_001129970.1
CYC-R	CCA GGT ACT CCA TCA GTG TCT CC	
CFLAR-F	CGA AGG ATG TTC ATG GCA GA	NM_001001628.1

CFLAR-R	CAC CTC CAG GAA GCT GCT GT	
CASP3-F	CGA GGC ACA GAA TTG GAC TG	NM_214131.1
CASP3-R	CCA GGA ATA GTA ACC AGG TGC TG	
CASP10-F	ACA GGA TGG ACC GGA ATC AC	NM_001161640.1
CASP10-R	CGC CTC AGA CAT TCA GCA TC	
PCNA-F	TCC TGA AGA AGG TGC TGG AA	NM_001291925.1
PCNA-R	GAG ACG AGT CCA TGC TCT GC	
CCND2-F	GCA GAA CTT GCT GAC CAT CG	NM_214088.1
CCND2-R	CAC AGA CCT CCA GCA TCC AG	
CCNA-F	GGC ACT GCT GCT ATG CTG TT	NM_001177926.1
CCNA-R	ACG AGG TGC TCC ATT CTC AA	
GADD45-F	GTG TCA GGA ATG CAG CGA CT	XM_005654701.2
GADD45-R	TTC CAG GCA TCT GTG TGA GG	
SOD2-F	AAC AAC CTG AAC GTC GTG GA	NM_214127.2
SOD2-R	GGC CTC CAC CGT TGA ACT T	
HADHA-F	AGC TCC GCA GAA GGA GGT TA	NM_213962.2
HADHA-R	GGT GCT TAT CCA CGG AGA CC	
ACADM-F	GAG GAG CCA TTG ATG TGT GC	NM_214039.1
ACADM-R	CGG ATC AGA ACG AGC CAA TA	
MYL9-F	CAA GGC CAA GAC CAC CAA G	NM_001244472.1
MYL9-R	CCA TCT CGG TTC TGG TCG AT	
ICAM1-F	TCA GAG GCT ACG GTC CAC CT	NM_213816.1
ICAM1-R	TGC TCC TTC TCG GTT CCA TT	
IFNG-F	TCT GCA GAT CCA GCG CAA AG	NM_213948.1
IFNG-R	GCT CTC TGG CCT TGG AAC AT	
CCL4-F	TCT GCG TGA CTG TCC TCT CC	NM_213779.1
CCL4-R	GCT TCC GCA CGG TGT ATG T	
IL-8-F	TCC AAA CTG GCT GTT GCC TT	NM_213867.1
IL-8-R	ACA GTG GGG TCC ACT CTC AA	
MAP2K1-F	TCG ATG AAC AGC AGC GGA AGC	NM_001143716.1
MAP2K1-R	ACC TTG AAC ACC ACA CCT CCA TTG	
β-actin-F	TCTGGCACCACACCTTCT	U07786.1
β-actin-R	TGATCTGGGTCATCTTCTCAC	

Supplemental Table 2: Primers for RT-PCR in this study.
